# Concentration-dependent oscillation of specific loss power in magnetic nanofluid hyperthermia

**DOI:** 10.1038/s41598-020-79871-1

**Published:** 2021-01-12

**Authors:** Ji-wook Kim, Jie Wang, Hyungsub Kim, Seongtae Bae

**Affiliations:** Nanobiomagnetics and Bioelectronics Laboratory (NB2L), Department of Electrical Engineering, University of South Carolina, 301 Main Street, Columbia, SC 29208 USA

**Keywords:** Chemistry, Engineering, Materials science, Nanoscience and technology, Physics

## Abstract

Magnetic dipole coupling between the colloidal superparamagnetic nanoparticles (SPNPs) depending on the concentration has been paid significant attention due to its critical role in characterizing the Specific Loss Power (SLP) in magnetic nanofluid hyperthermia (MNFH). However, despite immense efforts, the physical mechanism of concentration-dependent SLP change behavior is still poorly understood and some contradictory results have been recently reported. Here, we first report that the SLP of SPNP MNFH agent shows strong concentration-dependent oscillation behavior. According to the experimentally and theoretically analyzed results, the energy competition among the magnetic dipole interaction energy, magnetic potential energy, and exchange energy, was revealed as the main physical reason for the oscillation behavior. Empirically demonstrated new finding and physically established model on the concentration-dependent SLP oscillation behavior is expected to provide biomedically crucial information in determining the critical dose of an agent for clinically safe and highly efficient MNFH in cancer clinics.

## Introduction

Magnetic nanofluid hyperthermia (MNFH) has been paid significant attention as a potential treatment modality in cancer clinics due to its biotechnical advantages such as deep tissue penetration of AC magnetic heat induction power [specific loss power (SLP)] with minimal attenuation and unexpectedly lower “side effects”^1–3^ etc. Accordingly, a great deal of research activities to practically apply MNFH for cancer clinics including the design of high performance magnetic nanoparticles (MNPs) enabling to generate high SLP for completely killing tumors and the development of highly enhanced biotechnology to effectively improve the in-vitro/in-vivo biocompatibility etc., have been intensively conducted for the last two decades. As the result, it was demonstrated that the optimized selection of size, shape, and composition of MNPs is a crucial factor to improve the SLP, chemical, physical, biochemical, and magnetic characteristics of nanofluid agents for clinically safe MNFH applications^2,4,5^. Additionally, it was verified that the control of MNP’s surface and coating process conditions are critical for the enhancement of biocompatibility^6,7^.

Theoretically, SLP cannot be controlled by the concentration of MNFH agents, because it does not have any obvious dependence on the concentration^8,9^. However, according to several studies recently reported, it was surprisingly observed that the change of SLP has a dependence on the concentration of MNFH agent^10–21^. This observation is not only scientifically interesting but also medically important since this unexpected behavior can result in the severely wrong prediction of heat generation performance of MNFH agents in clinical applications. Therefore, some scientific efforts to interpret the unexpected concentration-dependent SLP change behavior have been intensively made for the recent few years. According to the results, it was understood that magnetic dipole interaction caused by MNPs in nanofluids with different interparticle distances, *d*_*c–c*_, could be the main reason for the unexpected phenomenon. The strong magnetic dipole interaction, i.e. supposed to be caused by a short interparticle distance, may result in the decrease of SLP due to a chain-like shaped arrangement of MNPs under AC magnetic field^10,11,14^. However, the physical mechanism of SLP change is still poorly understood, and some contradictory results have been recently reported^15,16^. This may be thought to be due to the variation of experimental conditions such as the magnetic nature of MNPs, the degree of colloidal stability, the condition of applied AC magnetic field, and the wrongly selected magnetic parameters in theoretical analysis. Moreover, owing to the tiny size and the dynamic motion of colloidal MNPs in water (nanofluids), it would be more difficult to physically interpret the nature of magnetic dipole interaction compared to typical solid systems such as magnetic thin film systems. Therefore, systematically well-designed direct/indirect experimental conditions of nanofluids, i.e. accurately controlled *d*_*c–c*_ with minimized aggregations, are essentially needed to systematically analyze the concentration-dependent magnetic dipole interaction behavior and interpret its physical effects on the change behavior of SLP.

In this study, *d*_*c–c*_-dependent magnetic dipole coupling energy induced in nanofluids and their physical contribution to the SLP change behavior were investigated and analyzed by measuring the intrinsic/extrinsic magnetic parameters of nanofluids as a function of concentration. Mg shallow doped γ-Fe_2_O_3_ superparamagnetic NPs (SPNPs) (Mg_x_–γFe_2_O_3_, *d* = 25 nm) with a narrow size distribution (< 10%) and a high colloidal stability were employed to measure the AC magnetic heat induction characteristics at the different concentrations varied from 0.12 mg_(Fe)_/mL to 40 mg_(Fe)_/mL. The heating-up rate, dT/dt, was determined from the AC heat induction curves for SLP calculation. To study the effects of magnetic dipole moment, *m*, of each Mg_x_–γFe_2_O_3_ SPNP on the physical characteristics of magnetic dipole interaction in Mg_x_–γFe_2_O_3_ SPNP nanofluid, the dependence of SLP on the concentration was investigated at the applied AC magnetic fields, *H*_*AC,appl*_, with a fixed frequency of *f*_*appl*_ = 100 kHz and the different field strength changed from 70 to 140 Oe. This is because the $\normalsize m$ of Mg_x_–γFe_2_O_3_ nanofluid is typically proportional to the strength of *H*_*AC,appl*_ at this range. Moreover, to further study the effects of $m$ on the magnetic dipole interaction and its induced magnetostatic energies, $E_{ms}$, different kinds of shallow doped SPNP nanofluids (Mg_x_–γFe_2_O_3_, Ni_x_Zn_1−x_–γFe_2_O_3_, and K_x_–γFe_2_O_3_ nanofluids) with different magnetization, M, were considered because M is defined as the volume density of *m*. To comprehensively understand the underlying physics of *d*_*c–c*_-dependent SLP change behavior and the induced $E_{ms}$ in SPNP nanofluids, concentration-dependent M, initial susceptibility, $\chi_{0}$, and coercivity, *H*_*c*_, were systematically measured at the *H*_*AC,appl*_/DC magnetic field, *H*_*DC,appl*_*,* using a vibrating-sample magnetometer (VSM) and a AC magnetic susceptometer. From all the experimental and analyzed results, a concrete physical model was built up and the concentration dependent-SLP change behavior was interpreted in terms of *d*_*c–c*_-induced competition of $E_{ms}$ in nanofluid for clinically safe MNFH applications.

## Result and discussion

### Theoretical background of magnetic dipole interaction between the colloidal SPNPs

**Figure 1:**
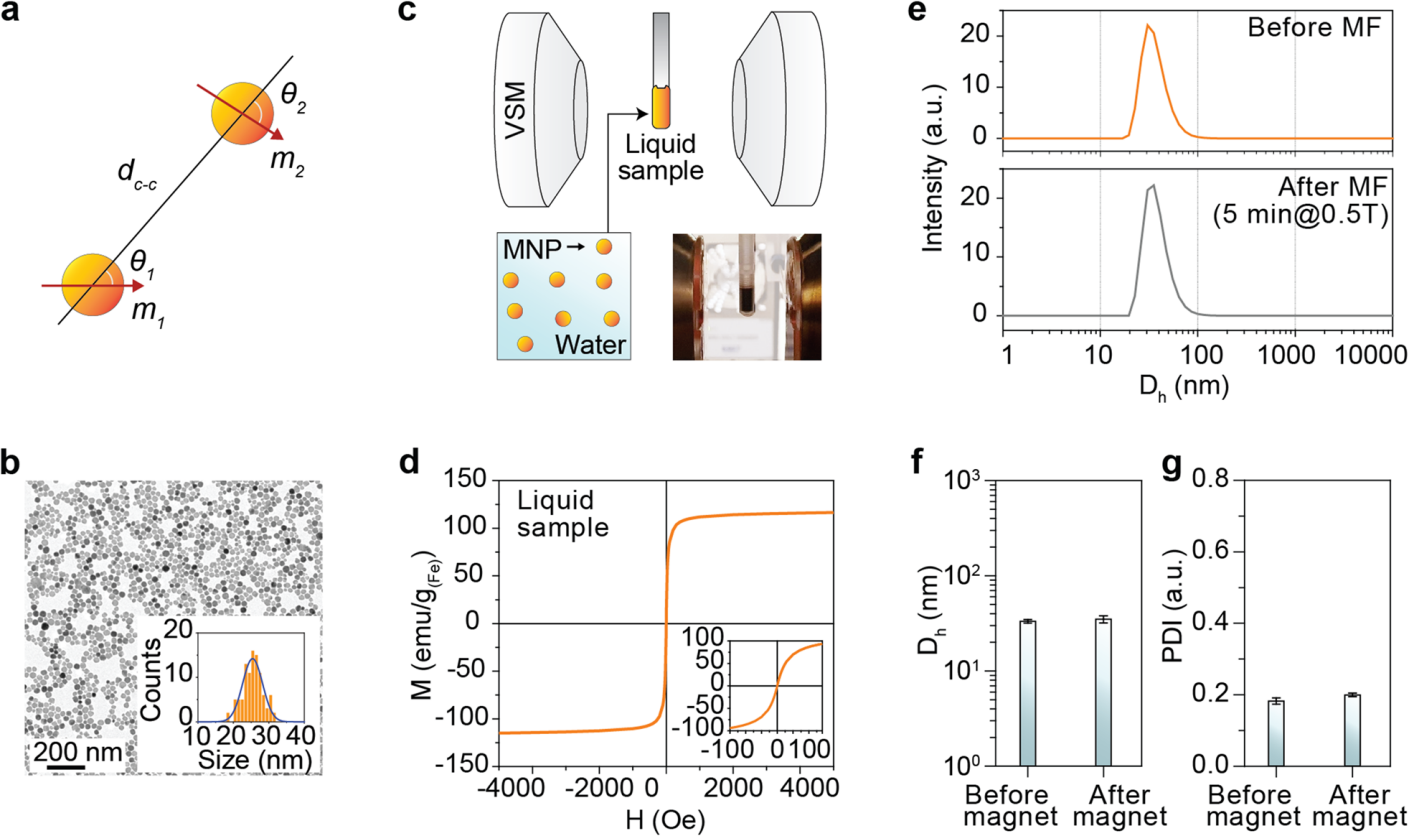
Dipole–dipole interaction model and nanofluid characterization. (**a**) A schematic model of dipole–dipole interaction between two SPNPs. *d*_*c–c*_, *m*, and *θ* represent center-to-center interparticle distance, the magnetic moment of each MNP, and angle between tow dipole induced by the SPNPs, respectively. (**b**) A TEM image of 25 nm Mg_x_–γFe_2_O_3_ SPNP (inset, the size distribution of Mg_x_-γFe_2_O_3_ SPNP measured from TEM image). (**c**) A liquid VSM measurement of magnetic properties of Mg_x_-γFe_2_O_3_ nanofluids. (inset, a picture of Mg_x_–γFe_2_O_3_ nanofluid sample mounted on the VSM holder). (**d**) M–H major loop of Mg_x_–γFe_2_O_3_ nanofluids measured at the sweeping field of ± 5 kOe (inset, minor M–H loop). (**e**) The hydrodynamic sizes before (top) and after (bottom) applied *H*_*DC,appl*_ (5 min@0.5 T). (**f**,**g**) Measured hydrodynamic size (**f**) and polydispersity index (PDI, **g**) of the nanofluids before and after the applied *H*_*DC,appl*_.

The mutual potential energy, which is a kind of “magnetostatic energy”, *E*_*dip*_, resulted from magnetic dipole interaction between two sphere SPNPs under an external magnetic field, is well-known to have a dependence on the *m* of each SPNP and the mean center-to-center *d*_*c–c*_. If each SPNP has *m* and the two SPNPs are separated by *d*_*c–c*_, the *E*_*dip*_ can be expressed by Eq. (1) based on the “*Stoner–Wöhlfarth model*”^12,13^.

1 $$E_{dip} = \frac{{m_{1} m_{2} }}{{d_{c - c}^{3} }}\left[ {\cos \left( {\theta_{1} - \theta_{2} } \right) - 3{\text{cos}}\theta_{1} {\text{cos}}\theta_{2} } \right],$$

where *θ*_1_ and *θ*_2_ are the angles between two magnetic dipoles as depicted in Fig. 1a. To investigate the contribution of magnetic dipole (interparticle) interaction induced by the SPNPs in nanofluid to the AC heat induction characteristics, the concentration-dependent *d*_*c–c*_ and the strength of *m* directly relevant to the magnetic anisotropy as well as the Nèel relaxation time, $\tau_{N}$ are considered as the most crucial parameters in analyzing the dipole interaction behavior and its induced $E_{ms}$ in nanofluids. The *d*_*c–c*_ between the SPNPs can be calculated by considering the number of particles in different concentrations of nanofluids to interpret the SLP change behavior^8,22^. Additionally, considering the Brownian relaxation time, $\tau_{B}$, given by Eq. (2), nanofluids with a narrow hydrodynamic size distribution along with minimized aggregation are inevitably required to obtain a uniform *m* and explore the effects of concentration-dependent change of effective hydrodynamic volume, $V_{h,eff}$, on the SLP for reliable interpretation. The $\tau_{B}$ is proportional to the $V_{h,eff}$, of the colloidal SPNPs.

2 $$\tau_{B} = \frac{{3\eta V_{h,eff} }}{{K_{B} T}},$$

where $K_{B}$ is Boltzmann constant, $T$ is the absolute ambient temperature in Kelvin, and *η* is the viscosity of nanofluids, respectively.

### Preparation and characterization of nanofluids

By considering all the physical, chemical, and magnetic experimental conditions preliminarily required, Mg_x_–Fe_2_O_3_ nanofluids with a narrower size distribution (25 ± 2.4 nm in diameter, Fig. 1b) were prepared as a testing sample. Another reason is because it has superior AC heat induction performance^2,23^ allowing for obvious observation of SLP change depending on the concentration. The detailed information on the structural, magnetic, and chemical properties of Mg_x_–γFe_2_O_3_ SPNPs and its nanofluids can be found in Supplementary Figs. 1a,b, and Refs. 2, and 23. Polyethylene glycol (PEG) was coated on the surface of Mg_x_–γFe_2_O_3_ SPNPs to fully prevent aggregation from *H*_*AC,appl*_ and *H*_*DC,appl*_ during measurement. A VSM measurement using a liquid sample was conducted to investigate the magnetic dipole (interparticle) interaction and the intrinsic/extrinsic magnetic properties of the nanofluids with different concentrations (Fig. 1c). The saturation magnetization, *M*_*s*_, of Mg_x_–γFe_2_O_3_ nanofluid was determined at a 110 emu/g_(Fe atom)_ (4.07 × 10^–15^ emu/particle@140 Oe) by DC M–H loop at the sweeping field of ± 5 kOe (Fig. 1d). The *H*_*c*_ obtained from the DC minor M-H loop (inset in Fig. 1d, sweeping field: ± 150 Oe) was smaller than 0.05 Oe indicating that it has superparamagnetic property with minimized aggregation. The hydrodynamic sizes before (Fig. 1e, top) and after applied *H*_*DC,appl*_ (5 min@0.5 T, Fig. 1e, bottom) were 31.3 nm, and 32.2 nm, respectively (Fig. 1f). The polydisperse index (PDI) were 0.18, and 0.19, respectively (Fig. 1g). All the results shown in Fig. 1 demonstrate that PEG-coated Mg_x_–γFe_2_O_3_ nanofluid is ideal to study the effects of magnetic dipole interaction on the SLP change behavior due to its superparamagnetic property, uniform size, and superior colloidal stability against the externally *H*_*AC,appl*_ and *H*_*DC,appl*_.

### Investigation on the concentration-dependent SLP change behavior

**Figure 2:**
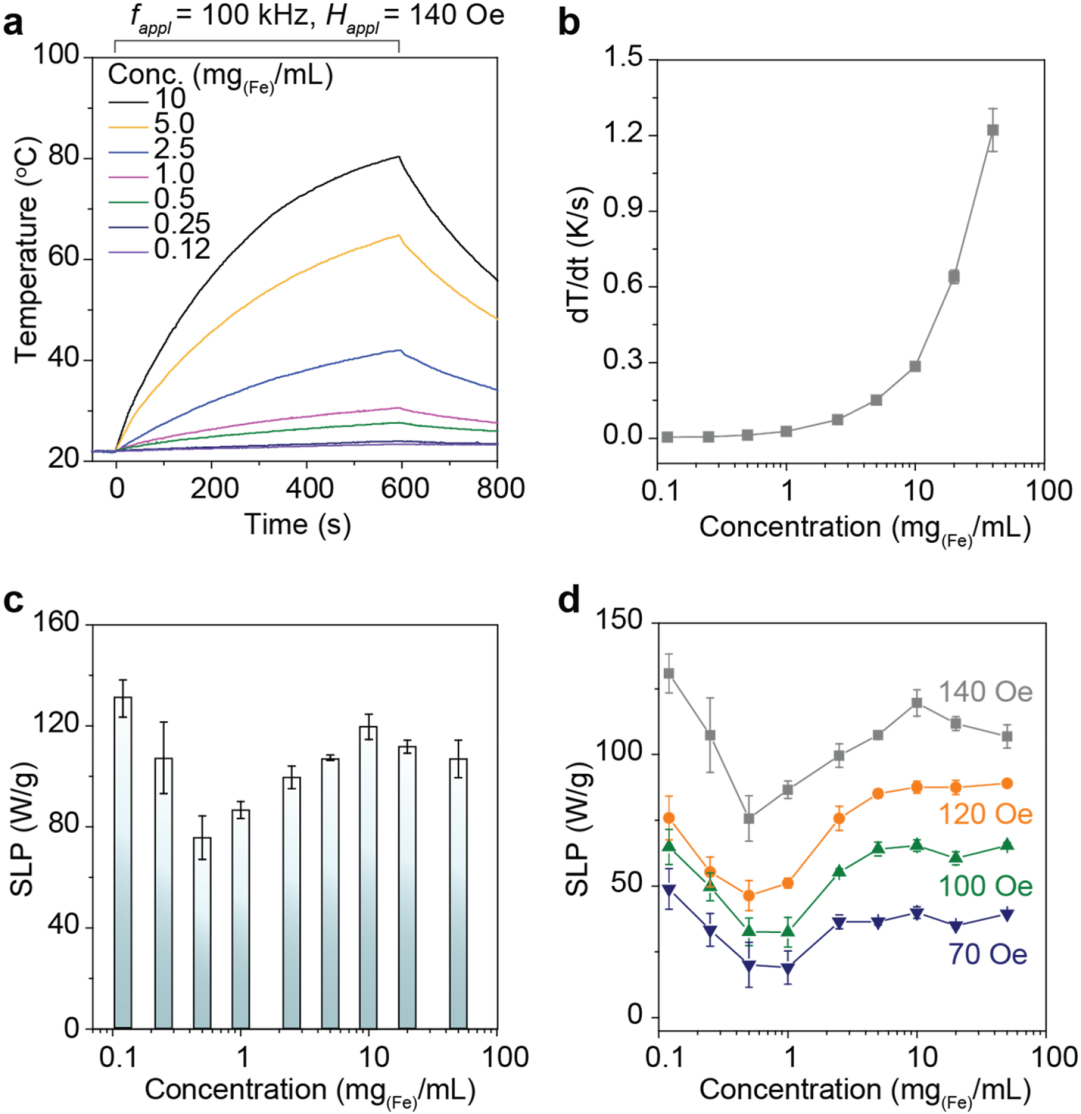
Concentration-dependent AC magnetic heat induction characteristics of Mg–γFe_2_O_3_ nanofluids. (**a**) AC heat induction curves of Mg_x_–γFe_2_O_3_ nanofluids with different concentrations (0.12 ~ 10 mg_(Fe)_/mL) measured at *H*_*AC,appl*_ of *f*_*appl*_ = 100 kHz and *H*_*appl*_ = 140 Oe. (**b**,**c**) Concentration-dependent dT/dt (heating up rate) (**b**) and SLP changes (**c**). (**d**) Concentration-dependent SLP changes measured at the different strength of *H*_*AC,appl*_ of 70 (blue), 100 (green), 120 (orange), and 140 Oe (gray).

To systematically explore the concentration-dependent SLP change behavior, Mg_x_–γFe_2_O_3_ SPNPs nanofluid with different concentrations, 0.12, 0.25, 0.5, 1.0, 2.5, 5.0, 10, 20, and 40 mg_(Fe)_/mL, were prepared. The volume was constant at 1 mL for all the experiments. Since the molecular weight of 25 nm Mg_x_–γFe_2_O_3_ SPNP core is 2.6 × 10^7^ g/mol, the estimated number of SPNPs in a 1 mL of solution is to be 4.5, 9.3, 18, 37, 93, 180, 370, 740, and 930 × 10^12^ particles, respectively. Assuming that individual SPNP is occupied in the same volume of solution, the *d*_*c–c*_ numerically calculated is to be 600, 480, 380, 300, 220, 175, 140, 110, and 90 nm, respectively. The AC magnetic heat induction was characterized at the biologically and physiologically safe range of *H*_*AC,appl*_ (*f*_appl_·*H*_appl_ < 5.0 × 10^9^ Am^−1^ s^−1^ or *f*_appl_ < 120 kHz, *H*_appl_ < 190 Oe)^24,25^ to investigate the effects of magnetic dipole (interparticle) interaction on the SLP change behavior. The *H*_*AC,appl*_ was fixed at a 100 kHz and the field strength was varied from 70 to 140 Oe. Figure 2a shows the AC heat induction curves of Mg_x_–γFe_2_O_3_ nanofluids with different concentrations (0.12 ~ 10 mg_(Fe)_/mL) measured at the fixed *H*_*AC,appl*_ of *f*_*appl*_ = 100 kHz and *H*_*appl*_ = 140 Oe. It was clearly observed that the temperature was proportionally increased up to 80 °C by increasing the concentration from 0.12 to 10 mg_(Fe)_/mL. The AC heat induction curves for the higher concentrations, i.e. 20 and 40 mg_(Fe)_/mL, was shown in Supplementary Fig. 2. The dT/dt determined from Fig. 2a is shown in Fig. 2b. The first 30 s of heat change (30 data points) was considered to calculate SLP for the reliable determination^26^. Figure 2c shows the dependence of concentration on the change of SLP. Similar to the previous reports^11,14,15^, the SLP was decreased from 135 to 75 W/g by increasing the concentration from 0.12 to 0.5 mg_(Fe)_/mL (380 < *d*_*c–c*_ < 600). As previously reported, this can be similarly understood that the appearance of magnetic dipole interaction induced by the *d*_*c–c*_ < 600 nm in Mg_x_–γFe_2_O_3_ nanofluids would be the primary physical/chemical reason for the obvious degradation. However, it was interesting that the SLP was increased and then decreased again, like an “*oscillation behavior*”, by further increasing the concentration. At a 0.5 ~ 40 mg_(Fe)_/mL (90 nm < *d*_*c–c*_ < 380 nm) range of concentration, the SLP was suddenly increased with a maximum value of 118 W/g (10 mg_(Fe)_/mL), and then it was decreased again. This oscillation behavior of SLP in a certain range of concentration is an uncommon physical phenomenon and has not been observed yet in MNFH studies. According to Eq. (1), *E*_*dip*_ must be theoretically proportional to $d_{c - c}^{3}$. However, SLP change behavior shown in Fig. 2c does not follow by Eq. (1). This indicates that not only *E*_*dip*_ but also other concentration-dependent or *d*_*c–c*_ dependent magnetostatic energies, *E*_*ms*_, which are competitive with *E*_*dip*_ activated by the *H*_*AC,appl*_ and *H*_*DC,appl*_, are associated with characterizing the SLP oscillation. To further investigate the physical nature of concentration-dependent “oscillation behavior” of SLP, the M of Mg_x_–γFe_2_O_3_ nanofluid was systematically controlled by applying different *H*_*AC,appl*_ of 70, 100, 120, and 140 Oe at the fixed frequency of *f*_*appl*_ = 100 kHz. The strength of *H*_*AC,appl*_ was varied within the saturation magnetic field of Mg_x_–γFe_2_O_3_ nanofluids (inset, Fig. 1d), because a linear and power-law relationship are co-existed in the between M and the *H*_*AC,appl*_. As can be seen in Fig. 2d, the concentration-dependent “oscillation behavior” of SLP become obvious by increasing the *H*_*AC,appl*_ strength. The concentration-dependent oscillation period of SLP change are very similar independent of *H*_*AC,appl*_, but the amplitude of local minimum and maximum SLP had a clear dependence on the strength of *H*_*AC,appl*_. This indicates that *E*_*dip*_ is closely related to the concentration-dependent “oscillation behavior” of SLP changes. Moreover, the slightly different appearance of SLP change at the different strength of *H*_*AC,appl*_ illustrates that not only *E*_*dip*_ but also other *E*_*ms*_ are involved in the “oscillation behavior” of SLP as described in Fig. 2c.

**Figure 3:**
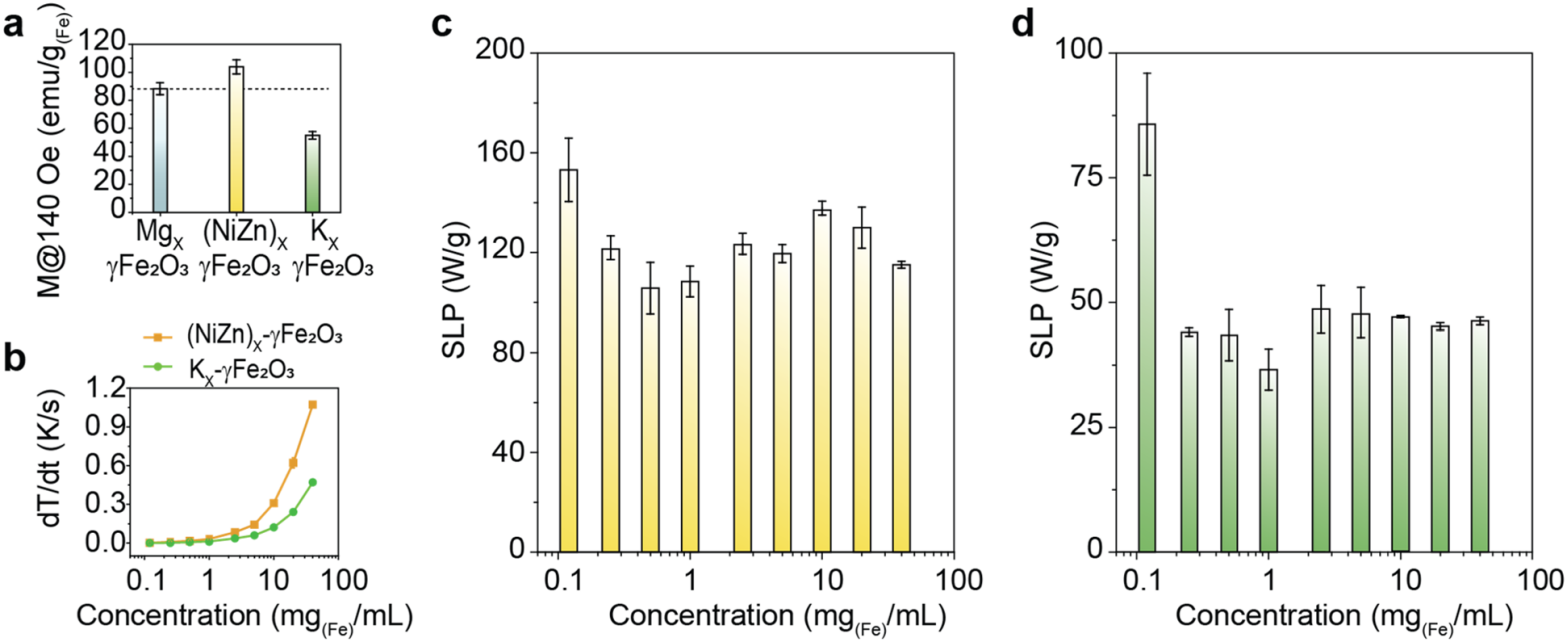
Effects of magnetization (M), volume density of magnetic dipole moment, of nanofluids on the concentration-dependent SLP changes and its behavior. (**a**) M of Mg_x_–γFe_2_O_3_, (NiZn)_x_–γFe_2_O_3,_ and K_x_–γFe_2_O_3_ nanofluids determined from M-H minor loops at the sweeping field of ± 140 Oe. (**b**) Concentration-dependent dT/dt of (NiZn)_x_–γFe_2_O_3_ and K_x_–γFe_2_O_3_ nanofluids. (**c**,**d**) Concentration-dependent SLP change behavior of (NiZn)_x_–γFe_2_O_3_ (**c**) and K_x_–γFe_2_O_3_ (**d**) nanofluids.

To further confirm the effects of strength of *m* on the concentration-dependent “oscillation behavior” of SLP change characteristics and to investigate what other *E*_*ms*_ could be involved in characterizing the “oscillation behavior”, well-designed MNFH experiments with different M values of nanofluids were conducted. As can be seen in Fig. 3a, 25 nm (NiZn)_x_–γFe_2_O_3_ and 25 nm K_x_–γFe_2_O_3_ nanofluids with a higher (120 emu/g_(Fe atom)_, 4.49 × 10^–15^ emu/particle@140 Oe), and a lower (50 emu/g_(Fe atom)_, 2.70 × 10^–15^ emu/particle@140 Oe) M value than that of Mg_x_–γFe_2_O_3_ nanofluids were used for comparison. Moreover, a 13 nm Mg_x_–γFe_2_O_3_ nanofluids (0.48 × 10^–15^ emu/particle@140 Oe) with a much smaller M value than that of 25 nm Mg_x_–γFe_2_O_3_ nanofluids were prepared (Supplementary Fig. 4a,b) for further investigation. As shown in Fig. 3b, (NiZn)_x_–γFe_2_O_3_ nanofluid had a higher dT/dt than that of K_x_–γFe_2_O_3_ nanofluid due to its higher M@ ± 140 Oe. The concentration-dependent AC heat induction characteristics and SLP change behavior (Fig. 3c,d, and Supplementary Fig. 3) of those two nanofluids were similar to those of Mg_x_–γFe_2_O_3_ nanofluids shown in Fig. 2a,d, respectively. Additionally, the SLP oscillation period of the two nanofluids were almost identical to the Mg_x_–γFe_2_O_3_ nanofluids, but the oscillation amplitude of SLP was different due to their different M@ ± 140 Oe values. It is interestingly noted that the SLP change behavior of K_x_–γFe_2_O_3_ nanofluids was also similar to that of Mg_x_–γFe_2_O_3_ nanofluids measured at the *H*_*AC,appl*_ of 70 Oe. This is thought to be due to the lower M@ ± 140 Oe (or relative magnetic hardness) of K_x_–γFe_2_O_3_ nanofluids. Similar oscillation behavior, i.e. smaller oscillation amplitude, was also observed in 13 nm Mg_x_–γFe_2_O_3_ nanofluids due to the lower M@ ± 140 Oe, but local SLP maximum peak at 10 mg_(Fe)_/mL was not observed (Supplementary Fig. 4c,d). Approximately ~ 10 times smaller M@ ± 140 Oe, i.e. 100 times smaller *E*_*dip*_, is thought to be the main reason for the disappearance of local SLP maximum peak at a higher concentration. All the results shown in Fig. 3 and Supplementary Figs. 3, 4, empirically demonstrate again that *E*_*dip*_ plays a crucial role in characterizing the concentration-dependent SLP “oscillation behavior”. However, as described in Eq. (1), since *E*_*dip*_ could be infinitely increased at a higher concentration due to the shorter *d*_*c–c*_, it is impossible to elucidate the SLP “oscillation behavior” with only *E*_*dip*_ at high concentrations. Therefore, other *E*_*ms*_, which can be generated by both concentration-dependent change of *d*_*c–c*_ in nanofluids and externally *H*_*AC,appl*_ and *H*_*DC,appl*_, such as (1) magnetic potential energy ($E_{p} = 2mHcos\theta )$^27^ directly related to magnetic stray field coupling energy and uniaxial anisotropy energy, and (2) exchange energy $(E_{ex} = - 2J_{ex} \overrightarrow {{S_{i} }} \cdot \overrightarrow {{S_{j} }} )$^27^, which is formed between the SPNPs (or two spins) and partially contribute to the magnetization reversal of spins in nanofluids, i.e. coherent or incoherent fanning mode, must be considered to reasonably and fully explain the SLP “oscillation behavior”.

### Magnetic analysis of concentration-dependent SLP “oscillation behavior”

**Figure 4:**
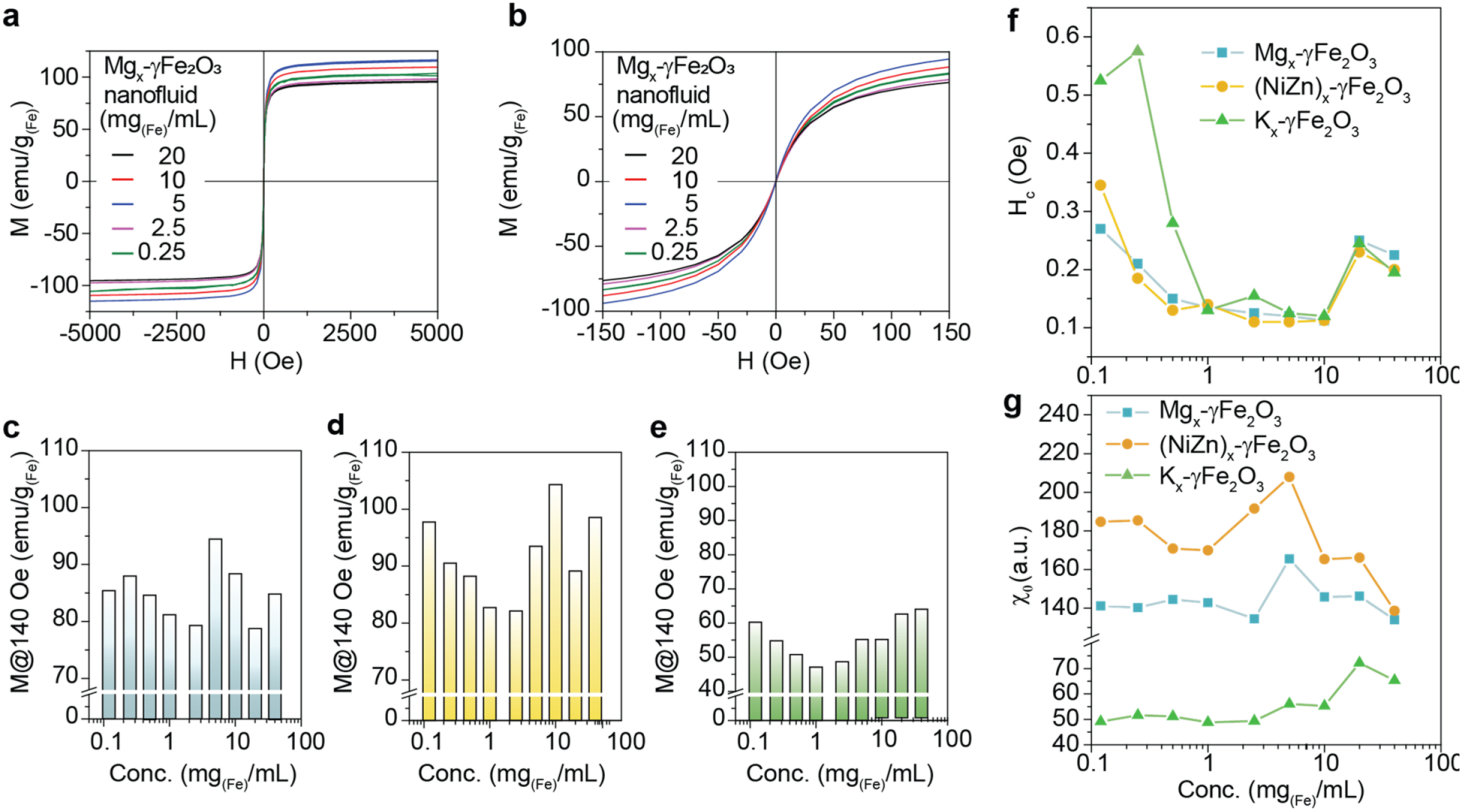
Magnetization (M), magnetic coercivity (*H*_*c*_), and magnetic initial susceptibility (χ_0_) of Mg_x_–γFe_2_O_3_, (NiZn)_x_–γFe_2_O_3_, and K_x_–γFe_2_O_3_ nanofluids. (**a**,**b**) Major (**a**) and minor (**b**) M–H loops of Mg_x_–γFe_2_O_3_ nanofluids with 0.25, 2.5, and 5 mg_(Fe)_/mL concentrations. (**c**–**e**) Concentration-dependent change behavior of M in Mg_x_–γFe_2_O_3_ (**c**), (NiZn)_x_–γFe_2_O_3_ (**d**), and K_x_–γFe_2_O_3_ (**e**) nanofluids. (**f**,**g**) Concentration-dependent change behavior of *H*_*c*_ (**f**) and χ_0_ (**g**) for Mg_x_–γFe_2_O_3_, (NiZn)_x_–γFe_2_O_3_, and K_x_–γFe_2_O_3_ nanofluids.

To deeply understand the nature of concentration-dependent SLP “oscillation behavior” and to study in details on the contribution of *E*_*ms*_ to the SLP oscillation characteristics, intrinsic and extrinsic magnetic parameters, i.e.* H*_*c*_, M@ ± 140 Oe (linear region), saturation magnetization (Supplementary Table 1), initial magnetic susceptibility, $\chi_{0}$, and AC hysteresis related to out-of-phase susceptibility, $\chi^{\prime\prime}$, of three nanofluids were measured using a VSM and an AC magnetosusceptometer. Figure 4 shows the intrinsic and extrinsic magnetic parameters of the nanofluids measured at the different concentrations varied in a range of 0.12 ~ 40 mg_(Fe)_/mL. As shown in Fig. 4a–e and Supplementary Fig. 5, the three nanofluids surprisingly exhibited very similar concentration-dependent M@ ± 140 Oe “oscillation behavior” to that of SLP. This indicates that the concentration-dependent SLP “oscillation behavior” is directly related to the M@ ± 140 Oe of the nanofluids. Furthermore, considering the relationship between M@ ± 140 Oe and *m* of each SPNP given in Eq. (1), it could be clearly thought that *E*_*dip*_ is primarily responsible for the concentration-dependent SLP “oscillation behavior”. As shown in Fig. 4f and Supplementary Fig. 6, the concentration-dependent *H*_*c*_ (an extrinsic magnetic parameter) had very similar change behavior to that of M@ ± 140 Oe and SLP. However, there is no close similarity in the between $\chi_{0}$ (an intrinsic magnetic parameter) and M@ ± 140 Oe change behavior. This analyzed result indirectly demonstrates that not only *E*_*dip*_ but also other *E*_*ms*_ participated in the magnetization reversal of spins in the SPNP nanofluids are closely related to the SLP “oscillation behavior”. Either coherent or incoherent spin reversal resulted from the concentration-dependent change of *d*_*c–c*_ can be responsible for both M@ ± 140 Oe and SLP “oscillation behavior” along with *E*_*dip*_. It is well known that the M, especially $\chi^{\prime\prime},$ measured by AC hysteresis at the *H*_*AC,appl*_ is proportional to the SLP, because the area of AC hysteresis is proportional to the $\chi^{\prime\prime}$^2,28,29^. To verify the coincident relationship between SLP and M($\chi^{\prime\prime})$ under *H*_*AC,appl*_, AC hysteresis was measured at the *H*_*AC,appl*_ of *f*_*appl*_ = 100 kHz and *H*_appl_ = 140 Oe, which is the same condition for the AC heat induction measurement. According to results, Mg_x_–γFe_2_O_3_ nanofluid had a larger AC hysteresis area than that of K–γFe_2_O_3_.

### Establishing a physical model to elucidate the concentration-dependent SLP “oscillation behavior”

**Figure 5:**
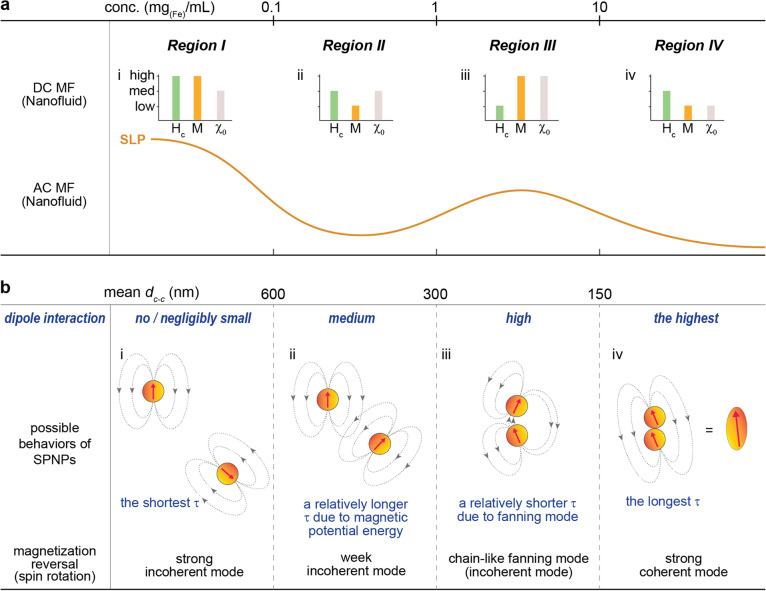
Physical model of particle interaction. A physical model of particle interaction as a function of the concentration of nanofluids during MNFH considering magnetic dipole interaction energy (*E*_*dip*_), magnetic potential energy (*E*_*p*_), magnetic exchange energy (*E*_*ex*_), and magnetization reversal of each nanoparticle under AC magnetic field by spin rotation enabling to elucidating concentration-dependent oscillation behavior of SLP changes. (**a**,**b**) Summary of experimental results (**a**) and possible particle interaction models (**b**) at the AC magnetic field interpreted by means of interparticle distance (*d*_*c–c*_).

Based on the analyzed AC/DC magnetic parameters of the three nanofluids at the different concentrations, a physical model, which can successfully elucidate the concentration-dependent SLP “oscillation behavior”, was proposed and built up as shown in Fig. 5. The model interprets the oscillation characteristics by dividing four specific regions depending on the concentrations: *Region I*: < 0.1 mg_(Fe)_/mL, *Region II*: 0.1 ~ 1.0 mg_(Fe)_/mL, *Region III*: 1.0 ~ 10 mg_(Fe)_/mL, and *Region IV*: > 10 mg_(Fe)_/mL. Additionally, it explains the physical principle of SLP “oscillation behavior” in terms of the energy competition between the concentration (*d*_*c–c*_)-dependent change of *E*_*dip*_ and interparticle distance-induced *E*_*ms*_ = *E*_*p*_ + *E*_*ex*_. The magnetization reversal of each SPNP in nanofluids by *H*_*AC,appl*_ and *H*_*DC,appl*_ is assumed to be spin rotation based on the “*Stoner-W*ö*hlfarth model*”^27^. At *Region I*, the lowest concentration (~ 0.1 mg_(Fe)_/mL, *d*_*c–c*_ > 600 nm), as shown in Fig. 5a(i), the nanofluids showed the highest SLP, the high (intrinsic) M, the largest *H*_*c*_, and medium (almost intrinsic) $\chi_{0}$. In this region, SPNPs in nanofluids are supposed to have randomly aligned spins due to the longest *d*_*c–c*_ expecting to lead no/negligibly small *E*_*dip*_ and no magnetic stray field coupling between the SPNPs caused by no induced *E*_*p*_ even at the *H*_*AC,appl*_ and *H*_*DC,appl*_. This makes the nanofluids have the largest *H*_*c*_ due to strong spin incoherency under *H*_*DC,appl*_, the medium (almost intrinsic) $\chi_{0}$, and the high (intrinsic) M. Figure 5b (i) shows a well-described schematic diagram for the lowest concentration of nanofluid. At *Region II* (0.1–1.0 mg_(Fe)_/mL, 300 nm < *d*_*c–c*_ < 600 nm), as can be seen in Figs. 5a (ii) and b (ii), the nanofluids exhibited a remarkable drop in M and SLP, a slight decrease in *H*_*c*_, and medium (almost intrinsic) $\chi_{0}$. The remarkable drop in M including $\chi^{\prime\prime}$ is thought to be due to the appearance of *E*_*dip*_ and the magnetic stray field coupling energy, *E*_*p*_, between or among the SPNPs under *H*_*AC,appl*_ and *H*_*DC,appl*_ caused by a shorter *d*_*c–c*_ compared to *Region I*. Additionally, the increase of $\tau_{N}$ due to the weak spin incoherency and the reduction of M induced by a shorter *d*_*c–c*_ as well as the increase of $\tau_{B}$ due to the possibly increased $V_{h,eff}$ of partially existed magnetically-coupled SPNPs caused by *E*_*p*_ are supposed to result in the drop in SLP. The physical relationship between the AC/DC M, especially $\chi^{\prime\prime}$ and the AC heat induction power directly related to SLP is well describe in Eq. (3)^2,8,30^.

3 $$P = \pi \mu_{0} H_{appl }^{2} f_{appl } \chi^{\prime\prime} = \pi \mu_{0} H_{appl }^{2} f_{appl} \chi_{0} \frac{{2\pi f_{appl } \tau }}{{1 + (2\pi f_{appl } \tau )^{2} }}$$

where *µ*_*0*_ is permeability in equilibrium condition, *χ*_*0*_ is equilibrium (or natural) susceptibility of SPNPs, and $\tau$ ($\frac{{1}}{\tau }{ = }\frac{{1}}{{{\uptau }_{{\text{N}}} }}{ + } \frac{{1}}{{{\uptau }_{{\text{B}}} }}$) is relaxation time constant. According to Eq. (3), a shorter *d*_*c-c*_ can be expected to induce a relatively weak incoherent mode of spin rotation during the magnetic reversal compared to *Region I*. Moreover, by combining with a widely used phenomenological expression for the coercivity given in Eq. (4)^31,32^, the slightly decreased *H*_*c*_ can be understood that it is due to the reduction of M dominantly caused by the generation of *E*_*p*_ and *E*_*dip*_ because the calculated *K*_*u*_ of solid Mg_x_–γFe_2_O_3_ nanoparticles using ZFC–FC measurement is constant at a $1.21 \times 10^{{4}} {\text{J/m}}^{{3}}$(data not shown).

4 $$H_{K} \left( {H_{c} } \right) = \alpha_{K} \frac{{2K_{u} }}{{\mu_{0} M_{s} }} - D_{eff} M_{s} - \Delta H(T,\eta )$$

where $\it {\upalpha }_{{\text{K}}}$ is the Kronmuller parameter, $\it {\text{D}}_{{{\text{eff}}}}$ is the a magnetic interaction parameter, $\it \Delta {\text{H}}$ is the fluctuation-field contribution caused by thermal activation, and $\eta$ is the the sweep rate (*dH/dt*). The negligibly small change of $\chi_{0}$ illustrates that a shorter *d*_*c–c*_-induced *E*_*dip*_ at *Region II* alone is not strong enough to change the intrinsic $\chi_{0}$ of the nanofluids. The more increase of nanofluids concentration will further develop *E*_*dip*_ and *E*_*p*_ together due to a much shorter *d*_*c–c*_. At the further shorter *d*_*c–c*_ (< 300 nm), it is expected to generate another *E*_*ms*_, [weak (short-range) or strong (long-range)] *E*_*ex*_, among the SPNPs in the nanofluids depending on *d*_*c–c*_. The energy competition of these three energies, *E*_*dip*_, *E*_*p*_, *E*_*ex*_, lead to make two different chain-like spin rotation modes, fanning incoherent mode and strongly coherent mode, in nanofluids (*Region III* and *Region IV*) under *H*_*AC,appl*_ and *H*_*DC,appl*_. At *Region III* (1.0–10 mg_(Fe)_/mL, 150 nm < *d*_*c–c*_ < 300 nm), as shown in Figs. 5a (iii) and b (iii), the SLP and M were interestingly re-increased and surprisingly $\chi_{0}$ was increased from intrinsic value. These interesting phenomena would be due to the formation of a chain-like incoherent fanning mode of spins in the adjacent SPNPs caused by the much shorter *d*_*c–c*_. Although *E*_*dip*_ is stronger compared to *Region II*, *E*_*p*_ along with weakly generated *E*_*ex*_ are comparable or larger than *E*_*dip*_ that is why a chain-like incoherent fanning mode of spins can be formed adjacent SPNPs. As shown in Fig. 5b (iii), the north and south poles are closer together, but *E*_*p*_ + *E*_*ex*_ > *E*_*dip*_. This causes the easy spin rotation of adjacent SPNPs due to a lowered total *E*_*ms*_ barriers under *H*_*AC,appl*_ and *H*_*DC,appl*_^27^. Accordingly, it leads to a decrease in *H*_*c*_, the large increase in M ($\chi^{\prime\prime}$), and the re-increase in SLP due to the faster $\tau_{N}$ and $\tau_{B}$ caused by the fanning mode of spin rotation compared to *Region II*. The increased $\chi_{0} = \chi_{0}^{intrinsic} + \chi_{0}^{extrinsic,increased}$ compared to *Regions I* and *II* demonstrate that a stronger *E*_*dip*_ and weakly formed *E*_*ex*_ resulted from the much shorter *d*_*c-c*_ is existed in this range of concentration. Moreover, from the physical definition, $\chi_{0} = \chi_{0}^{^{\prime}}$(in-phase)$+ \chi_{0}^{^{\prime\prime}}$(out-of-phase), it can be understood that a stronger *E*_*dip*_ and weakly formed *E*_*ex*_ induced increase of $\chi^{\prime\prime}$ is another physical evidence for the re-increase of SLP. At *Region IV* (the highest concentration and the shortest *d*_*c–c*_ (> 10 mg_(Fe)_/mL, *d*_*c–c*_ < 150 nm), the SLP and M were re-decreased and $\chi_{0}$ was strangely decreased as shown in Fig. 5a (iv). The *E*_*dip*_ and *E*_*p*_ are expected to be maximized and the *E*_*ex*_ will be minimized in this region due to the shortest *d*_*c–c*_. Therefore, the highest *E*_*dip*_ and *E*_*p*_ make all the spins of adjacent SPNPs coherently align in the nanofluids and can lead to form prolate spheroids due to strong spin coherency as illustrated in Fig. 5b (iv). The coherently aligned spins in chain-likely contacted SPNPs will produce a large magnetostatic stray field. This can demagnetize adjacent SPNPs (or prolate spheroids). Additionally, the long-chain like SPNPs (or prolate spheroids) can generate themselves a shape anisotropy-induced demagnetizing field in the nanofluids at the steady state/non-steady state conditions. These cause the re-reduction of M and correspondingly the re-increase of *H*_*c*_ when they were exposed to the *H*_*AC,appl*_ and *H*_*DC,appl*_ for magnetic reversal and AC magnetic excitation. Accordingly, the SLP was re-decreased due to the reduction of *M* ($\chi^{\prime\prime}$) (or increased AC magnetic hardness) under *H*_*AC,appl*_. In addition, the existence of a prolate spheroid type of SPNPs (SPNPs clusters) in the nanofluids makes the $V_{h,eff}$ much larger causing the decrease in SLP due to the longer $\tau_{B}$. The decrease of $\chi_{0}$ is thought to be attributed to the increase of DC/AC magnetic hardness of the nanofluids resulted from the stronger *E*_*dip*_ and *E*_*p*_ induced by the shortest *d*_*c–c*_.

## Conclusion

It was first observed that the AC heat induction power (SLP) of SPNP MNFH agent exhibits strong concentration-dependent oscillation behavior. According to the experimentally and theoretically analyzed results, it was demonstrated that not only generally well-known *E*_*dip*_ but also the other two *E*_*ms*_ , i.e. *E*_*p*_ and *E*_*ex*_, activated by the concentration-dependent change of *d*_*c–c*_ under the *H*_*AC,appl*_ and *H*_*DC,appl*_ are directly involved in the SLP oscillation behavior. Moreover, the concentration-dependent energy competition among the *E*_*dip*_, *E*_*p*_, and *E*_*ex*_ was revealed as the main physical reason for the SLP oscillation. The empirically demonstrated new finding and physically established model on the concentration-dependent SLP oscillation behavior is expected to provide biomedically and technically crucial information in determining the bioavailable critical dose of agent for clinically safe and highly efficient MNFH in the future nanomedical cancer clinics. According to the experimentally demonstrated results, the ideal concentration of nanofluid for efficient MNFH will be a 5 ~ 10 mg_(Fe)_/mL locating at the second maximum peak of SLP because the temperature increase of 0.12 mg_(Fe)_/mL nanofluid (< 2 °C) is not sufficiently enough for killing the cancer tumors.

## Methods

### Preparation of nanofluids

For the synthesis of Mg_x_-γFe_2_O_3_ MNP, Mg acetate tetrahydrate (0.13 mmol), Fe acetylacetonate (2.0 mmol), oleic acid (1.2 mmol), and benzyl ether (20 mL) were mixed and magnetically stirred in a 250 mL round-bottom flask. The mixed reaction solutions were heated up to 200 °C for 30 min (~ 8 °C /min, the first ramping up rate) and maintained for another 60 min under N_2_ gas at the flow rate of ~ 100 mL/min. Then, the solutions were heated again up to 300 °C for 30 min (~ 5 °C /min, the second ramping rate) and maintained for 60 min. After removing the heat source, the product was cooled down to room temperature. For the water dispersion of pre-synthesized particles, Mg_x_–γFe_2_O_3_ MNP was coated with PEG. 50 mg of Mg_x_–γFe_2_O_3_ MNP was dissolved in 5 mL of 0.8 M TMAOH-Methanol solution. Then, 1.5 mL of 2-[methoxy(polyethyleneoxy)_9–12_ propyl] trimethoxysilane (m.w. = 591–723 g/mol) was added to the mixture solution and sonicated for 8 h at 70 °C. After the sonication, PEG-coated Mg_x_–γFe_2_O_3_ MNP was collected with NbFeB magnet and the supernatant was discarded. Collected PEG-coated Mg_x_–γFe_2_O_3_ MNP was rinsed 2 times with 20 mL of toluene and acetone, respectively. Finally, PEG-coated Mg_x_–γFe_2_O_3_ MNP was dispersed in 5 mL of deionized water. Ni/Zn- and K-doped γ-Fe_2_O_3_ MNPs were prepared using the same protocol.

### AC heat induction and SLP measurement of Nanofluids

AC Heat induction of nanofluid was characterized using an AC magnetic field induction system consisting of AC coils, capacitors, DC power supplies, and wave generators. The tubes containing nanofluids (1 mL) were placed in the center of AC coil. The *f*_*appl*_ was fixed at 100 kHz for this study. *H*_*AC*__,__*appl*_ was controlled from 70 to 140 Oe. The temperature of the nanofluid was measured by a fiber-optic thermometer (sampling rate: 1 point/s). The SLP values of all the nanofluids were calculated based on the following equation.

$${\text{SLP [W}}\,{\text{g}}^{ - 1} {] = }\frac{{{\text{C}}V_{s} }}{{\text{m}}}\frac{dT}{{dt}}$$

(C is the volumetric specific heat capacity, *V*_*s*_ is the sample volume, m is the a mass of magnetic material, *dT/dt* is the initial slope of the graph of the change in temperature versus time). *dT/dt* was obtained from temperature changes during the first 30 s of the heating curve.

### VSM measurement of nanofluids

To measure a DC hysteresis loop of nanofluids, an 80 μL of nanofluid (concentration: 0.12 ~ 40 mg_(Fe)_/mL) was loaded to the VSM sample holder and a cap was closed carefully to avoid air bubbles. Then, the sample was mounted to VSM. To test magnetic colloidal stability of MNPs under DC magnetic field, 5000 Oe DC magnetic field was applied to sample for 5 min. After the application of magnetic field, the hydrodynamic size was measured. Major and Minor hysteresis loop of nanofluids were measured at the sweeping field of ± 140 Oe and ± 5000 Oe, respectively. All measurement and analysis protocols were basically the same as the typical VSM measurement method.

## Supplementary Information

Supplementary Information.

## References

[1] Johannsen M (2007). Thermotherapy of prostate cancer using magnetic nanoparticles: Feasibility, imaging, and three-dimensional temperature distribution. Eur. Urol..

[2] Jang JT (2018). Giant magnetic heat induction of magnesium-doped γ-Fe_2_O_3_ superparamagnetic nanoparticles for completely killing tumors. Adv. Mater..

[3] Jordan A, Scholz R, Wust P, Fähling H, Felix R (1999). Magnetic fluid hyperthermia (MFH): Cancer treatment with AC magnetic field induced excitation of biocompatible superparamagnetic nanoparticles. J. Magn. Magn. Mater..

[4] Sharifi I, Shokrollahi H, Amiri S (2012). Ferrite-based magnetic nanofluids used in hyperthermia applications. J. Magn. Magn. Mater..

[5] Jeun M, Moon SJ, Kobayashi H, Shin HY, Tomitaka A (2010). Effects of Mn concentration on the ac magnetically induced heating characteristics of superparamagnetic Mn_x_Zn_1−x_Fe_2_O_4_ nanoparticles for hyperthermia Related Articles. Cit. Appl. Phys. Lett.

[6] Nel AE (2009). Understanding biophysicochemical interactions at the nano-bio interface. Nat. Mater..

[7] Xie J, Liu G, Eden HS, Ai H, Chen X (2011). Surface-engineered magnetic nanoparticle platforms for cancer imaging and therapy. Acc. Chem. Res..

[8] Rosensweig RE (2002). Heating magnetic fluid with alternating magnetic field. J. Magn. Magn. Mater..

[9] Fortin JP (2007). Size-sorted anionic iron oxide nanomagnets as colloidal mediators for magnetic hyperthermia. J. Am. Chem. Soc..

[10] Wang A, Li J, Gao R (2009). The structural force arising from magnetic interactions in polydisperse ferrofluids. Appl. Phys. Lett..

[11] Branquinho LC (2013). Effect of magnetic dipolar interactions on nanoparticle heating efficiency: Implications for cancer hyperthermia. Sci. Rep..

[12] Ilg P (2017). Equilibrium magnetization and magnetization relaxation of multicore magnetic nanoparticles. Phys. Rev. B.

[13] Ota S, Takemura Y (2019). Characterization of Néel and Brownian relaxations isolated from complex dynamics influenced by dipole interactions in magnetic nanoparticles. J. Phys. Chem. C.

[14] Serantes D (2010). Influence of dipolar interactions on hyperthermia properties of ferromagnetic particles. J. Appl. Phys..

[15] Haase C, Nowak U (2012). Role of dipole-dipole interactions for hyperthermia heating of magnetic nanoparticle ensembles. Phys. Rev. B Condens. Matter Mater. Phys..

[16] Martinez-Boubeta C (2012). Adjustable hyperthermia response of self-assembled ferromagnetic Fe-MgO core-shell nanoparticles by tuning dipole-dipole interactions. Adv. Funct. Mater..

[17] Jeun M (2009). Effects of particle dipole interaction on the ac magnetically induced heating characteristics of ferrite nanoparticles for hyperthermia. Appl. Phys. Lett..

[18] Urtizberea A, Natividad E, Arizaga A, Castro M, Mediano A (2010). Specific absorption rates and magnetic properties of ferrofluids with interaction effects at low concentrations. J. Phys. Chem. C.

[19] Dutz S, Hergt R (2012). The role of interactions in systems of single domain ferrimagnetic iron oxide nanoparticles. J. Nano-Electron. Phys..

[20] Ovejero JG (2016). Effects of inter- and intra-aggregate magnetic dipolar interactions on the magnetic heating efficiency of iron oxide nanoparticles. Phys. Chem. Chem. Phys..

[21] Conde-Leboran I (2015). A single picture explains diversity of hyperthermia response of magnetic nanoparticles. J. Phys. Chem. C.

[22] Frenkel YI (1955). Kinetic Theory of Liquids.

[23] Jang JT, Bae S (2017). Mg shallow doping effects on the ac magnetic self-heating characteristics of γ-Fe_2_O_3_ superparamagnetic nanoparticles for highly efficient hyperthermia. Appl. Phys. Lett..

[24] Bae S (2009). AC magnetic-field-induced heating and physical properties of ferrite nanoparticles for a hyperthermia agent in medicine. IEEE Trans. Nanotechnol..

[25] Hergt R, Dutz S, Müller R, Zeisberger M (2006). Magnetic particle hyperthermia: Nanoparticle magnetism and materials development for cancer therapy. J. Phys. Condens. Matter.

[26] Wildeboer RR, Southern P, Pankhurst QA (2014). On the reliable measurement of specific absorption rates and intrinsic loss parameters in magnetic hyperthermia materials. J. Phys. D. Appl. Phys..

[27] Cullity BD, Graham CD (2008). Introduction to Magnetic Materials.

[28] Youssif, M. I., Bahgat, A. A. & Ali, I. A. AC Magnetic susceptibility technique for the characterization of high temperature superconductors. *Egypt. J. Sol.* **23**, 231–250 (2000).

[29] Raikher, Y. L., Stepanov, V. I. & Perzynski, R. Dynamic hysteresis of a superparamagnetic nanoparticle. In *Physica B: Condensed Matter* vol. 343 262–266 (North-Holland, 2004).

[30] Hilger I (1998). Physical limits of hyperthermia using magnetite fine particles imaging therapeutic efficacy of magnetic hyperthermia view project physical limits of hyperthermia using magnetite fine particles. IEEE Trans. Magn..

[31] Kronmüller H (1987). Theory of nucleation fields in inhomogeneous ferromagnets. Phys. Status Solidi.

[32] Kronmüller H, Durst KD, Sagawa M (1988). Analysis of the magnetic hardening mechanism in RE-FeB permanent magnets. J. Magn. Magn. Mater..

