# Publisher Correction: Concentration-dependent oscillation of specific loss power in magnetic nanofluid hyperthermia

**DOI:** 10.1038/s41598-021-87308-6

**Published:** 2021-04-06

**Authors:** Ji-wook Kim, Jie Wang, Hyungsub Kim, Seongtae Bae

**Affiliations:** Nanobiomagnetics and Bioelectronics Laboratory (NB2L), Department of Electrical Engineering, University of South Carolina, 301 Main Street, Columbia, SC 29208 USA

Correction to: *Scientific Reports* 10.1038/s41598-020-79871-1, published online 12 January 2021

In the original PDF version of this Article, Equation 4 was omitted. This has now been corrected in the PDF; the HTML version of the paper was correct from the time of publication.

