# Supplementary material for: Concentration-dependent oscillation of specific loss power in magnetic nanofluid hyperthermia
Source: Sci Rep. 2021 Jan 12;11:733. doi: 10.1038/s41598-020-79871-1 (PMC7804452; doi:10.1038/s41598-020-79871-1)
Supplement: Supplementary file 1 — Supplementary Information. [file 41598_2020_79871_MOESM1_ESM.pdf]

Supplementary Information

Title

Concentration-dependent oscillation of specific loss power in magnetic nanofluid hyperthermia

Authors

Ji-wook Kim[1], Jie Wang[1], Hyungsub Kim[1], and Seongtae Bae[1,*]

[1]Nanobiomagnetics and bioelectronics laboratory (NB^{2}L), Department of Electrical Engineering, University of South Carolina, 301 Main Street, Columbia, SC 29208, USA

*Corresponding author

E-mail: bae4@cec.sc.edu

Materials and apparatus

Most of the materials (Mg acetate tetrahydrate, Ni acetate tetrahydrate, Zn acetate tetrahydrate, K acetate hydrate, Fe acetylacetonate, Tetramethylammonium hydroxide pentahydrate, oleic acid, oleyl amine, benzyl ether, methanol) that required for the synthesis of γ-Fe_2O_3 MNPs were purchased from Sigma Aldrich. PEG was purchased from Gelest. For the purification of MNPs, Amicon® centrifugal filter was purchased from MilliporeSigma. All glassware was obtained from KJ LAB (Seoul, Republic of Korea). For the heat induction measurement, AC magnetic field generator (NEO-NANOMEDIC, INC.) and fiber optic thermometer (OPTOCON®) were utilized. VSM (Lakeshore®) was used for DC magnetization measurement of MNPs.

X-ray diffraction patterns of Mg_x-γFe_2O_3 MNP

The crystal structure was analyzed using a Cu-Kα radiated X-ray diffractometer. All the synthesized Mg_x-γFe_2O_3, K_x-γFe_2O_3, and $(Ni/Zn)_x$-γFe_2O_3MNP showed a single-phase cubic spinel ferrite structure and did not exhibit any undesirable crystalline phases. All the X-ray diffraction patterns of Mg_x-γFe_2O_3 MNPs were well indexed and correlated to those of typical cubic spinel structures (JCPDS #38-0430).

X-ray absorption near edge structure (XANES) analysis

We have performed Fe K-edge XANES analysis for Mg_x-γFe_2O_3 MNPs to determine the local atomic structure. Fe K-edge X-ray absorption spectra were recorded on the BL10C beamline

of the Pohang light source II (PLS-II) with a ring current of 360 mA at 3.0 GeV under top-up operation. Si (111) double crystal monochromator has been employed to monochromatize the X-ray photon energy. The incident and transmitted X-ray photon flux were monitored with N2 gas-filled ionization. The EXAFS data from the samples were collected under the transmittance mode. Higher-order harmonic contaminations were eliminated by detuning to reduce the incident X-ray intensity by a ~30 %. Energy calibration has been simultaneously carried out for the measurement with a Fe metallic film placed in front of the third ion chamber. Fourier transform (FT) peak feature of Mg_x-γFe_2O_3 MNPs showed the typical radial distribution function of γ-Fe_2O_3 (maghemite). The decrease in the FT peak intensity (O_h-T_d corner shaped) can be attributed to the evolution of Fe defect site (for example, iron vacancy site) by the occurrence of Fe^{3+} ions.

Calculation of molecular weight of nanoparticles and concentration dependent d_{c-c}

In our previous publication, the Mg^{2+} ions doping concentration in iron oxide nanoparticles were determined using energy-dispersive X-ray spectroscopy (EDS) and an elemental mapping technique (Jang et al., Adv. Mater., 2017, 170462). From this analysis, the x value in Mgx-γFe_2O_3 nanoparticles was determined at a 0.13, which is very small value. Since we generally followed the same synthetic procedures (*i.e.*, the molar ratio between Mg/Ni-Zn/K and Fe source) to prepare all the three nanofluids, we believed that the doping level of Mg/Ni-Zn/K would be very small. Therefore, the molecular weight of all the three nanoparticles (2.59×10^7 g/mol) was calculated based on its size (25 nm) and using the typical density of Fe_2O_3 (*i.e.*, 5.24 g/cm^3). Based on the molecular weight and concentration of these nanoparticles, the number of nanoparticles in the unit volume was calculated. When we assume that the nanoparticles are

homogeneously distributed and fixed in the given space, the mean distance between the nanoparticles can be calculated depending on the nanofluid concentration.

Supplementary Figures

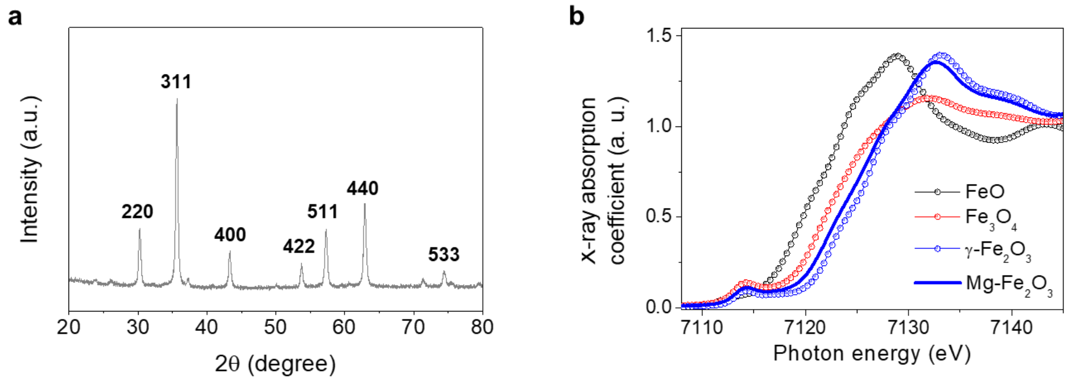

Supplementary Figure 1. Crystal structure analysis of Mg_x- γFe_2O_3 SPNP. **(a)** XRD result shows a conventional spinel structure. **(b)** XANES result shows that γ-phase structure of Mg_x- γFe_2O_3 SPNPs. (grey dot, FeO; red dot, Fe_3O_4; blue dot, γ-Fe_2O_3; blue line, Mg_x-γFe_2O_3).

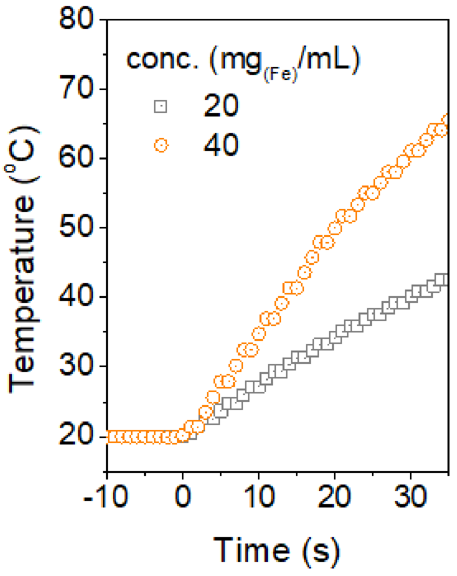

Supplementary Figure 2. Initial temperature changes of 20 (gray) and 40 $mg_{(Fe)}$/mL (orange) Mg_x-γFe_2O_3 nanofluid at AC magnetic field (f_{appl}: 100 kHz, H_{appl}: 140 Oe).

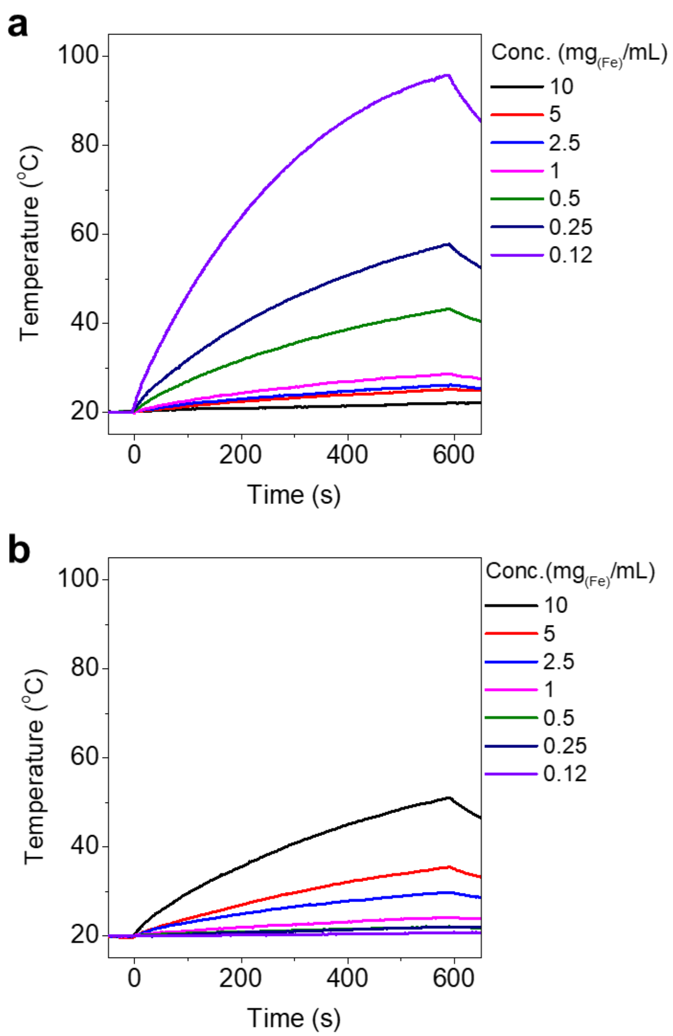

Supplementary Figure 3. Concentration-dependent AC magnetic heat induction characteristics. **(a,b)** Concentration-dependent AC magnetic heat induction characteristics of $(NiZn)_x$-γFe_2O_3 MNP nanofluids **(a)** and K_x-γFe_2O_3 MNP nanofluids **(b)**. Applied f_{appl} and H_{appl} of AC magnetic field were 100 kHz and 140 Oe, respectively.

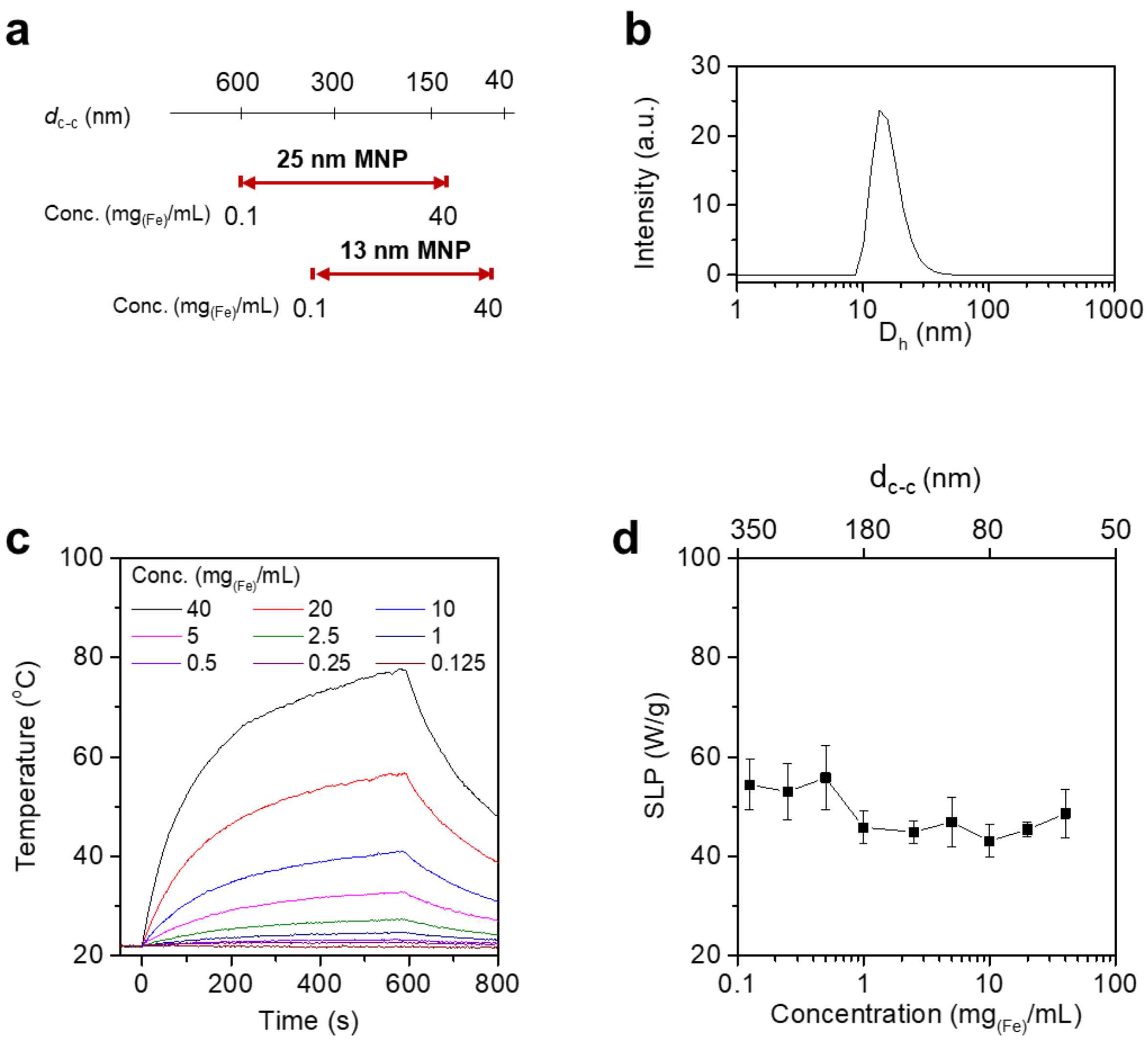

Supplementary Figure 4. Concentration-dependent AC magnetic heat induction characteristics of 13 nm Mg_x-γFe_2O_3 nanofluids. **(a)** Calculated concentration-dependent $d_{c\text{-}c}$ of 25 nm and 13 nm Mg_x-γFe_2O_3 nanofluids. **(b)** Hydrodynamic size of 13 nm Mg_x-γFe_2O_3 nanofluids. **(c,d)** Concentration-dependent AC magnetic heat induction characteristics **(c)** and their SLP changes **(d)** of 13 nm Mg_x-γFe_2O_3 nanofluids.

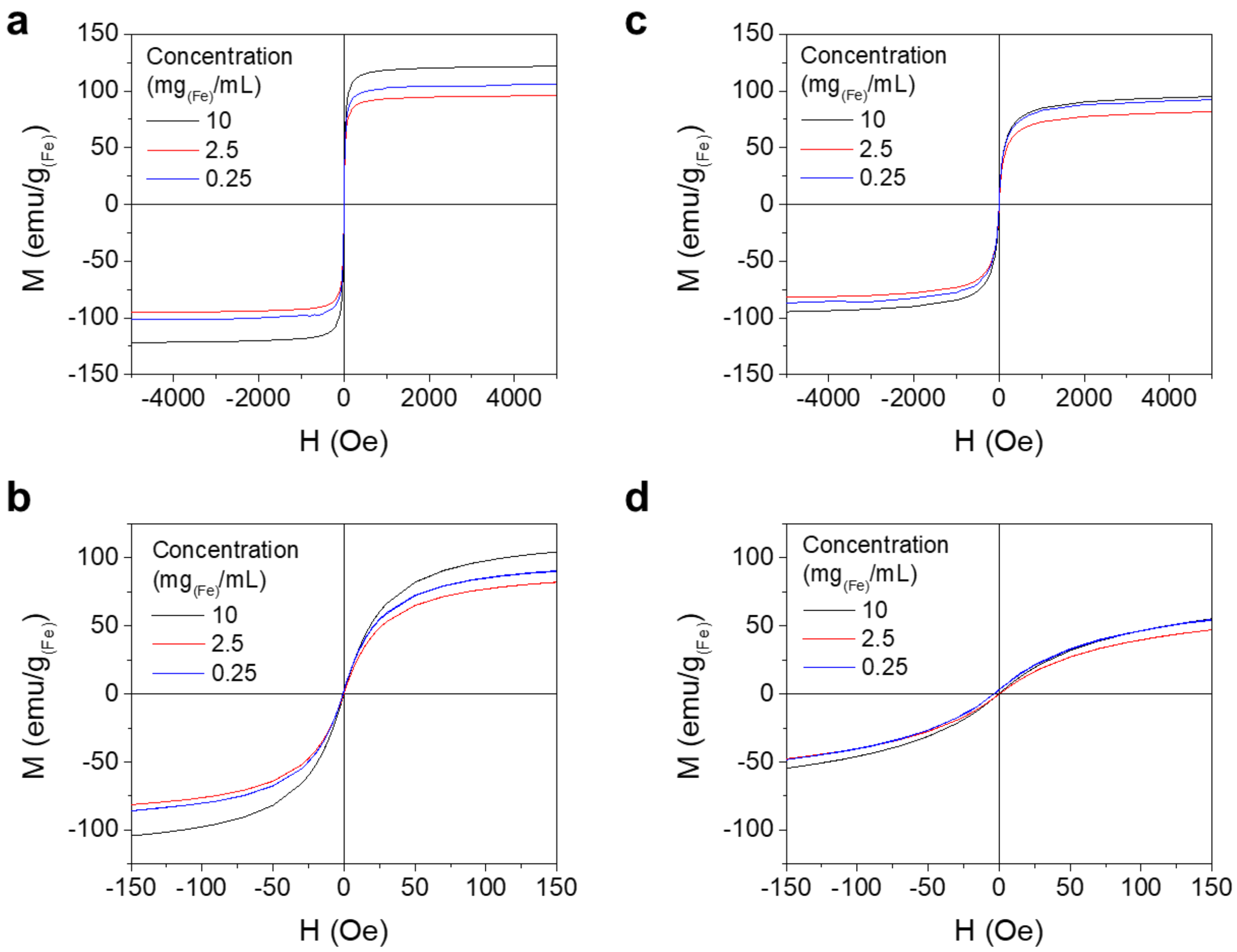

Supplementary Figure 5. M-H loops of $(NiZn)_x$-γFe_2O_3 and K_x-γFe_2O_3 nanofluids measured at the concertation of 0.25, 2.5, and 10 $mg_{(Fe)}$/mL. **(a,b)** Major **(a)** and minor **(b)** M-H loop of $(NiZn)_x$-γFe_2O_3 nanofluid. **(c,d)** Major **(c)** and minor **(d)** M-H loop of K_x-γFe_2O_3 nanofluid.

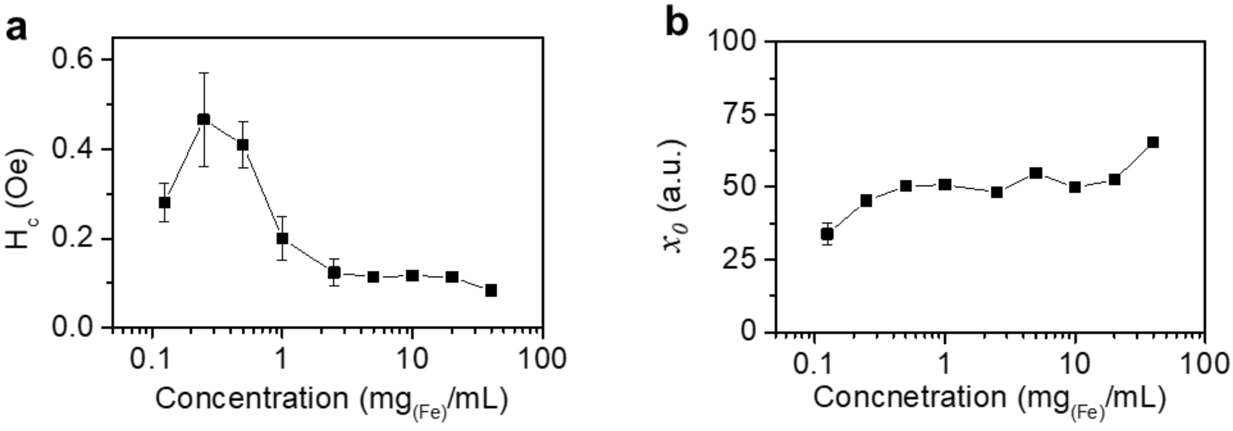

Supplementary Figure 6. Concentration-dependent magnetic behaviors of 13 nm Mg_x-γFe_2O_3 nanofluids. **(a,b)** Concentration-dependent change behavior of H_c **(a)** and χ_0 **(b)** of 13 nm Mg_x-γFe_2O_3 nanofluids.

Concentration ($mg_{(Fe)}$/mL)	Saturation magnetization (emu/$g_{(Fe)}$)		
	**Mgx-γFe_2O_3**	**Ni_xZn_{1-x}-γFe_2O_3**	**K_x-γFe_2O_3**
0.12	104.9	115.4	99.0
0.25	107.2	106.2	92.2
0.5	104.1	103.1	87.6
1	99.6	96.4	81.6
2.5	98.2	95.8	83.7
5	116.5	109.1	95.0
10	109.75	121.8	93.8
20	96.2	104.3	107.6

Supplementary Table 1. Saturation magnetization of Mgx-γFe_2O_3, Ni_xZn_{1-x}-γFe_2O_3, and K_x-γFe_2O_3 nanofluids.
